# Psychopathy and Depression: The moderating role of Psychopathic Personality Traits between Emotional Competence and Cognitive Functioning

**DOI:** 10.1192/j.eurpsy.2023.759

**Published:** 2023-07-19

**Authors:** E. M. D. Schönthaler, K. Schwalsberger, B. Reininghaus, E. Z. Reininghaus, N. Dalkner

**Affiliations:** Department of Psychiatry and Psychotherapeutic Medicine, Medical University of Graz, Graz, Austria

## Abstract

**Introduction:**

Psychopathic personality traits (PPT) are known to deteriorate emotional and cognitive functions, however, little is known about their role in depression. Nevertheless, depressive symptoms have also shown to be associated with emotional problems and worse cognitive functions and could thus also interact with PPT.

**Objectives:**

This study aimed to set up an integrative model by examining the correlative relationships and moderating role of PPT in the association between emotional competence and cognitive functioning in individuals with depression.

**Methods:**

Data from 373 individuals diagnosed with depression (158 males, 215 females) were investigated. Subjects filled out questionnaires surveying PPT and emotional competences. Furthermore, a comprehensive neuropsychological test battery investigating the cognitive domains Attention/Psychomotor Speed, Executive Functions and Verbal Learning/Memory was administered.

**Results:**

Correlation analyses revealed a significant positive association between emotional competence and overall cognitive functioning. Further, negative associations between emotional competence and the PPT “Blame Externalisation” and “Careless Nonplanfulness”, as well as positive associations with psychopathic “Social Potency” and “Stress Immunity” were found. Moderation analyses indicated a significant positive influence of psychopathic “Stress Immunity” and “Social Influence” on the relationship between emotional competence and cognitive parameters.

**Conclusions:**

The findings highlight the importance of considering PPT in further research on depression and reflect their impact in therapeutic settings.

**Disclosure of Interest:**

None Declared

